# Time trends in herpesvirus seroepidemiology among Swedish adults

**DOI:** 10.1186/s12879-024-09155-w

**Published:** 2024-03-02

**Authors:** Jan Olsson, Sema Nourmohammadi, Emma Honkala, Anders Johansson, Göran Hallmans, Bodil Weidung, Hugo Lövheim, Fredrik Elgh

**Affiliations:** 1 Department of Clinical Microbiology, Umeå University, Umeå, Sweden; 2 Department of Odontology, Umeå University, 901 97 Umeå, Sweden; 3 Department of Public Health and Clinical Medicine, Umeå University, Umeå, Sweden; 4 Department of Public Health and Caring Sciences, Clinical Geriatrics, Uppsala University, Uppsala, Sweden; 5 Department of Community Medicine and Rehabilitation, Geriatric Medicine, Umeå University, Umeå, Sweden

**Keywords:** Herpes, Herpes simplex, Epstein-Barr virus, Cytomegalovirus, Seroprevalence, Epidemiology, Time trends, Immunoglobulin G

## Abstract

**Background:**

Human herpesviruses are widespread among the human population. The infections often occur unnoticed, but severe disease as well as long-term sequelae are part of the symptom spectrum. The prevalence varies among subpopulations and with time. The aim of this study was to describe the seroprevalence of Immunoglobulin G against *Herpes simplex 1*, *Herpes simplex 2*, Epstein-Barr virus and Cytomegalovirus in the adult Swedish population over a time period of several decades.

**Methods:**

Serum samples (*n* = 892) from biobanks, originating from 30-year-old women, 50-year-old men and 50-year-old women sampled between 1975 and 2018, were analyzed for presence of anti-herpesvirus antibodies. Linear regression analysis was used to test for a correlation between birth year and seroprevalence. Multiple linear regression analysis was used to differentiate between other factors such as age and gender.

**Results:**

Birth year correlated negatively with the prevalence of immunoglobulin G against *Herpes simplex 1* and Epstein-Barr virus (*p* = 0.004 and 0.033), and positively with Immunoglobulin G against Cytomegalovirus (*p* = 0.039). When participant categories were analyzed separately, birth year correlated negatively with the prevalence of Immunoglobulin G against *Herpes simplex 1 and Herpes simplex 2* (*p* = 0.032 and 0.028) in 30-year-old women, and with the prevalence of Immunoglobulin G against Cytomegalovirus in 50-year-old men (*p* = 0.011).

**Conclusions:**

The prevalence of Immunoglobulin G against *Herpes simplex 1*, *Herpes simplex 2* and Epstein-Barr virus decreases in later birth cohorts. This indicates a trend of declining risk of getting infected with these viruses as a child and adolescent.

## Background

Human herpesvirus (HHV1-8) are globally distributed among the human population, causing a variety of diseases. Albeit phylogenetically closely related, the primary infections caused by the different HHVs are diverse both in symptoms spectra and the interplay in a wider sense between virus and human host. HHV-1&2 (HSV-1&2) cause oral and genital herpes with typical lesions, HHV-3 (VZV) cause chickenpox and shingles, HHV-4 (EBV) cause infectious mononucleosis, HHV-6&7 are the causative agents for roseola. Congenital infections of HHV-2 and HHV-5 (CMV) poses a high risk for the developing fetus [1] Typical for infections by HHV are the lifelong carriership once infected together with episodes of reactivated infection [1]. Several HHVs are linked to cancer; carriership of EBV drastically increase the risk for lymphoma and for carcinoma in the nasopharynx region [2], and HHV-8 is strongly associated with Kaposi´s sarcoma [3]. In addition to the well-characterized symptoms associated with primary infections and reactivations, several HHVs have been attributed roles in the development of chronic neurological disorders, most prominently represented by HSV-1 for Alzheimer’s disease and EBV- for Multiple Sclerosis [4–7]. Several HHVs may also act in concert to alter the disease trajectory, especially CMV and HHV-6 have been suggested such adjuvant roles [4, 8, 9]. When studied from an epidemiological perspective, factors such as geographic location, socioeconomic status and age influence the rate of acquisition of HHV infection [1]. In addition, host factors such as comorbidities, coinfections and immunosuppression influence both acquisition, reactivation and severity of symptoms [10–12] As most herpesvirus infections never end up in medical records, the detection of anti-HHV Immunoglobulin G (IgG) is currently the gold standard when screening for a history of HHV infection. An individual might be carrier of anti-HHV IgG (positive) or not (negative) and the proportion of positive individuals in a population is denoted seroprevalence. Well-grounded estimates of seroprevalence are helpful not only when assessing impact from herpesvirus infections on present and future neurological disease burden, but also to motivate vaccine development, and to guide decision-making regarding screening programs and treatment recommendations for a population. Among the HHVs, HHV-3 (VZV), HHV-6 A&B, and HHV-7 exhibit a seroprevalence close to 100% [1]. For VZV does a proportion of seropositive cases stem from vaccination. We performed the present study to estimate the seroprevalence of HHV-1 (HSV-1), HHV-2 (HSV-2), HHV-4 (EBV) and HHV-5 (CMV) over several decades in healthy adults from northern Sweden.

## Methods

With the aim to estimate time trends in seroprevalence of HHV-1 (HSV-1), HHV-2 (HSV-2), HHV-4 (EBV) and HHV-5 (CMV), serum from biobanks were analysed for presence of anti-HHV IgG with ELISA. By including participants of uniform age born between 1937 and 1988, seroprevalence in adults as a function of year of birth was monitored. Linear regression analyses were used to test for correlation.

### Participants

Samples were retrieved from two separate biobanks; The Northern Sweden Maternity cohort and The Västerbotten Intervention Programme / Northern Sweden Health and Disease Study Cohort (VIP/NSHDS) cohort. The Northern Sweden Maternity cohort consists of serum samples collected in conjunction with screening for infectious agents among pregnant women in their first trimester. The biobank includes samples from four counties (Västernorrland, Jämtland, Västerbotten and Norrbotten) in northern Sweden from 1975 and onward. Approximately 2400 samples are added each year, and at the time of sample retrieval for this study the maternity biobank comprised of approximately 102,000 individuals [13]. The VIP/NSHDS cohort consists of samples from residents in Västerbotten County. People at the age of 40, 50 and 60 are since 1985 invited to contribute a plasma sample in connection with health surveys for risk factor screening performed by the healthcare region of Västerbotten, Sweden [14]. At the time of sample retrieval consisted the biobank of 110,663 individuals.

The samples that were used from the maternity cohort are dated from 1975 to 2018 and from VIP/NSHDS 1987 to 2017. The range of year-of birth thus spans between 1937 and 1988, allowing for representation from birth cohorts that have entered a geriatric stage in life, as well as representation from relatively young individuals. Previous studies have indicated that span to cover a significant shift in seroprevalence, at least for anti-HSV-1 IgG [15]. Both cohorts are administered by The Biobank Research Unit at Umeå University, and are- by agreement with the healthcare region- made accessible for such research that serves the public health of the population. Integrity of the cohorts regarding IgG analyses have been proven earlier [16, 17]. For each sampling year, 12 serum samples from 30 year old women from the maternity cohort and 6 plasma samples each from 50 year old men and 50 year old women from the VIP/NSHDSkohort were retrieved in a randomized manner,. From the first year of the maternity cohort (1975) were only four samples possible to retrieve. A total of 892 samples were included.

### Serum and plasma analyses

Enzyme-Linked Immunosorbent Assay (ELISA) was used to detect anti-HSV-1 IgG, anti-HSV-2 IgG, anti-EBV IgG, and anti-CMV IgG.

ELISA assays deployed were, for anti-HSV-1 IgG and anti-HSV-2 IgG: HerpeselectⓇ-assays (Focus Diagnostics), for anti-EBV IgG: VCA IgG (VIROTECH Diagnostics), and for anti-CMV IgG: an in-house ELISA based on total tissue culture antigen, as previously described in [18]. Thresholds for a sample to be considered positive is for the HerpeSelectⓇ (anti-HSV-1 IgG, anti-HSV-2 IgG): 0.9X index value, for anti-EBV IgG more than 9.0 VE and for anti-CMV IgG more than 5U. Samples in the greyzone (*n* = 5–9) were considered positive, the rational being that this is biobank samples stored for a long time, and the chance is higher that some IgG is lost than the opposite have happened. The HerpeSelectⓇ-assays are claimed by the manufacturer to exhibit a sensitivity for HSV-1 / HSV-2 of 91/96% and a specificity of 92/97% (HerpeSelect® 1 ELISA IgG REF EL0910G Rev. K product package insert). Independent assessments have reported 70/92% sensitivity and 92/57% specificity [19], and 99/97% sensitivity, 77/89% specificity [20] when compared to western blot. The anti-EBV IgG assay has, in an early assessment, been reported to generate 2.7% false positives and 7.9% false negatives [21]. The performance of the in-house method was assured through external quality programs managed by Equalis (www.equalis.se/en/), UK Neqas (www.ukneqas.org.uk) and Lab-Quality (www.labquality.com). In addition, internal controls consisting of previously run sera were included in every test.

### Statistics

The seroprevalence was calculated by dividing the number of positive samples by all samples for each cohort. A linear regression analysis was used to test for a correlation between birth year and seroprevalence for the three groups separately: 30 year old women, 50 year old women and 50 year old men. A multiple linear regression analysis was used to differentiate between other factors and included overall prevalence in relation to birth year, age and gender.

*P* < 0.05 was regarded as statistically significant. The IBM SPSS 25 software for Mac was used for statistical calculations.

## Results

Three groups were studied separately: 30 years old women, 50 years old women and 50 years old men. Table 1 shows the background characteristics and seropositivity for each group.

**Table 1:** Background characteristics

	Females age 30 y	Males age 50 y	Females age 50 y
*n*	520	186	186
Birth years	1945 – 1988	1937 – 1967	1937 – 1967
Sampling years	1975 – 2018	1987 – 2017	1987 – 2017
Anti-HSV-1 IgG positive *n* (%)	350 (67.3)	141 (75.8)	137 (73.7)
Anti-HSV-2 IgG positive *n* (%)	77 (14.8)	22 (11.8)	35 (18.8)
Anti-EBV IgG positive *n* (%)	507 (97.5)	180 (96.8)	185 (99.5)
Anti-CMV IgG positive *n* (%)	382 (73.5)	143 (76.9)	153 (82.3)

*Abbreviations: n* number, *y* years, *HSV-1 Herpes simplex 1*, *IgG* Immunoglobulin G, *HSV-2 Herpes simplex 2*, *EBV* Epstein-Barr virus, *CMV* Cytomegalovirus

The relationship between birth year and seroprevalence was investigated for each of the three groups (Table 2). Statistically significant correlations were found between birth year and decreasing prevalence of anti-HSV-1 IgG (*p* = 0.032) and anti-HSV-2 IgG (*p* = 0.028) among 30-year-old women, and for anti-CMV IgG among 50-year-old men (*p* = 0.011).

**Table 2:** Herpes virus seroprevalence by birth year – linear regression analysis

		Female 30 y	Male 50 y	Female 50 y
Anti-HSV-1 IgG positive	Birth year beta	-0.004	-0.003	-0.007
	Birth year *p*-value	0.032	0.413	0.053
	Constant	7.603	6.399	14.380
Anti-HSV-2 IgG positive	Birth year beta	-0.003	-0.002	0.004
	Birth year *p*-value	0.028	0.480	0.234
	Constant	5.537	3.791	-7.289
Anti-EBV IgG positive	Birth year beta	-0.001	-0.002	-0.0003
	Birth year *p*-value	0.115	0.166	0.578
	Constant	2.672	4.903	1.651
Anti-CMV IgG positive	Birth year beta	0.002	-0.009	-0.004
	Birth year *p*-value	0.195	0.011	0.240
	Constant	-3.220	17.954	8.038

In a multiple linear regression analysis including overall seroprevalence in relation to birth year, age and female sex, birth year correlated negatively with the seroprevalence of anti-HSV-1 IgG (*p* = 0.004) and anti-EBV IgG (*p* = 0.033), while age correlated positively with anti-CMV IgG (*p* = 0.039) (Table 3).

**Table 3:** Herpes virus seroprevalence by birth year, age and sex – multiple linear regression analysis

	Anti-HSV-1 IgG positive	Anti-HSV-2 IgG positive	Anti-EBV IgG positive	Anti-CMV IgG positive
Birth year beta (*p*-value)	-0.004 (0.004)	-0.002 (0.103)	-0.001 (0.033)	-0.0002 (0.875)
Age beta (*p*-value)	0.0003 (0.898)	0.001 (0.679)	0.0003 (0.695)	0.004 (0.039)
Female sex beta (*p*-value)	-0.022 (0.648)	0.070 (0.059)	0.027 (0.080)	0.054 (0.225)

In the Female age 50 y group, there was a positive correlation between being anti-HSV-1 IgG positive and anti-CMV IgG positive (0.233, *p* = 0.001) and a negative correlation between being anti-HSV-1 IgG positive and anti-HSV-2 IgG positive (-0.149, *p* = 0.042). No other correlations were seen between antibody presence in any group.

## Discussion

Seroprevalence trends of anti-HSV-1 IgG, anti-HSV-2 IgG, anti-EBV IgG and anti-CMV IgG were studied in a population of 30-year-old pregnant women and 50-year-old men and women from northern Sweden. Although the vast majority of seroconversions for the studied HHVs take place in childhood and early adolescence, some increase in seroprevalence, as a consequence of de novo infection of adults, is predicted as a population is ageing [15]. Correct prediction of seroconversion rates requires longitudinal samples, a research asset not commonly available. In a multivariate regression analysis, we could confirm a significant increase of anti-CMV IgG seropositivity as a function of age, likely due to seroconversion in adult years. This is in line with previous reports [15, 22, 23].

**Fig. 1:**
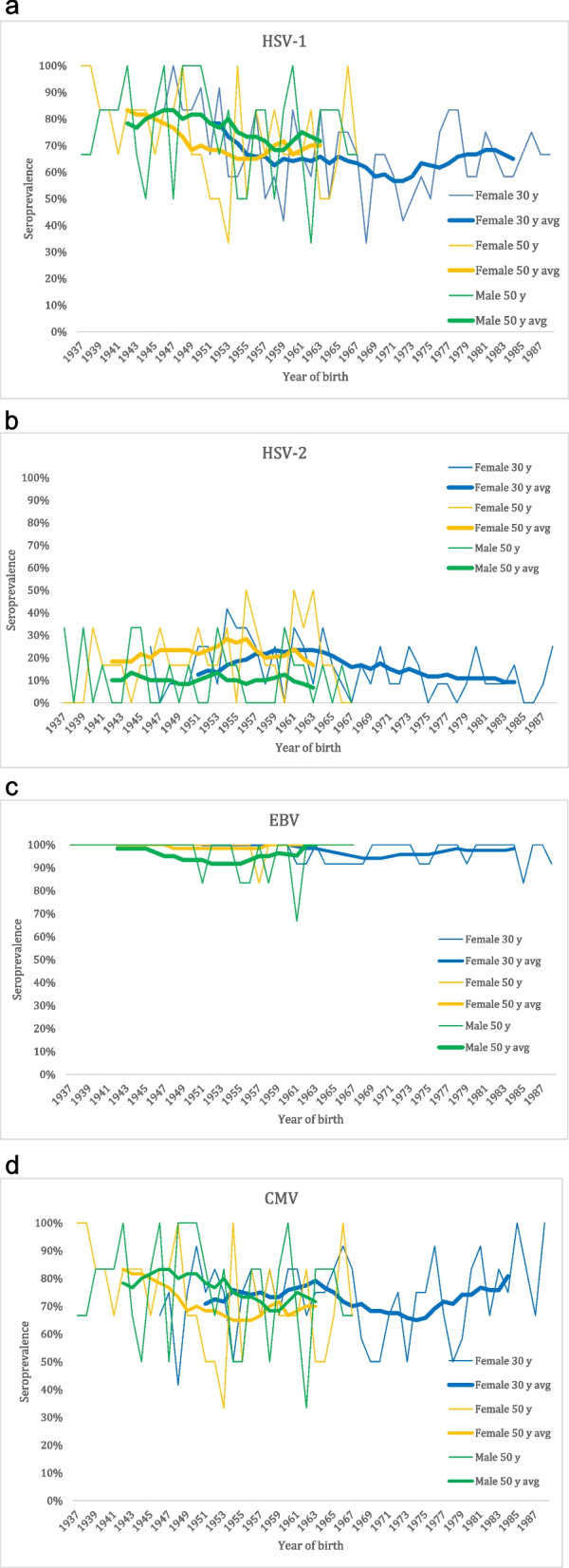
Seroprevalence of anti-HHV IgG among subjects born between 1937 and 1988. **a** represents anti-HSV-1 IgG, **b** represents anti-HSV-2 IgG, **c** represents anti-EBV IgG, **d** represents anti-CMV IgG. Subjects are divided into females aged 30 years (blue), females aged 50 years (yellow), and males aged 50 years (green). Thick lines represent the moving average over 10 years

For groups combined, later birth year correlated with lower seroprevalence for anti-HSV-1 IgG and anti-EBV IgG. For groups studied separately, later birth year correlated with lower seroprevalence for anti-HSV-1 IgG and anti-HSV-2 IgG among 30-year-old women, and with lower seroprevalence for anti-CMV IgG among 50-year-old men. The observed decrease in anti-HSV-1 IgG seropositivity for 30-year-old women is dramatic, from around 88% among women born in 1945–1948 to 69% among women born 1985–1988. This is in line with figures reported by other studies from Sweden [15], Finland [24], England [25] and the United States [26]. The diagram depicting a moving average over 10 years (Fig. 1a) suggests that the main part of the drop in anti-HSV-1 IgG seropositivity occurred between birth years 1950 and 1960, a time when the studied population proportionally changed from a rural to a more urban lifestyle. Interestingly, after that shift, the trend does not seem to continue. Speculatively, in the 1970s, daycare service and pre-schools with large groups of children were implemented on a large scale, a milieu notorious for transmission of infectious agents. The moving average curve for anti-HSV-2 IgG suggests a peak in seroprevalence for birth year around 1960, followed by a significant decrease (Fig. 1b). Other cohorts exhibit a similar major difference between birth givers born around 1960 and those born a few years later [27]. The decline for anti-HSV-2 IgG follows a trend shared by several sexually transmitted diseases (STDs) [28]. This effect have been attributed changes in sexual behavior, not to the least necessitated by the HIV pandemic [28]. The overall declining trend is in line with results from other studies [24, 26, 29]. The introduction of acyclovir as a suppressive treatment option for HSV infections in the mid-1980s is presumed to have reduced the rate of reactivation and changed the pattern of spread [30], as have the fact that HSV-1, instead of HSV-2, by now is the primary cause of genital HSV infections at least in some populations [31]. Public health implications of the decrease in seroprevalence include that a larger proportion of women enter pregnancy seronegative, and thus run a risk of contracting the infection and transferring it to the fetus.

EBV is a very common infection in our population, where over 96% were seropositive in this study. The declining trend in relation to birth year is statistically significant, but so small that implications for populational health are questionable (Fig. 1c). In a study from Finland, no declining trend in seroprevalence of EBV could be detected [24].

Comparing the moving average curves for anti-CMV IgG (Fig. 1d) and anti-HSV-1 IgG (Fig. 1a) gives an illustration of the similarities in spread between the two agents, which is further supported by the correlation between having these two agents among 50-year-old females. Interestingly, both anti-HSV-1 IgG and anti-CMV IgG seem to have their minimum around birth year 1970–1980. Further studies should investigate if the trend of increasing seroprevalence in later birth years is statistically significant.

### Strengths and weaknesses of the study

The limited information on each sample does not permit for detailed suggestions of underlying causes and correlations of the reported trends such as comorbidities, rates of reactivations, disease outcomes and other clinical data.. Also, the small number of samples from each year make yearly prevalence estimates as well as trends for sub-timespans uncertain. Therefore, to equalize random year-to-year variation we also present the 10-year moving average. A weakness of the study is that data for females originates from two separate cohorts, while data for males originates from one single cohort. This must be considered when comparisons are made. In addition, the 30-year old women are all pregnant, a fact that probably skews the selection, Strengths of the study include the employment of population-based biobanks [13], that allows for estimation of 50-year long time trends in age- and gender specific anti-HHV IgG prevalence among persons born between 1937 and 1988. The population-based biobanks represent an unselected sample of the underlying general population, with minimal risk of further selection bias apart from the above mentioned. This makes it likely that the observed trends do reflect the actual population prevalence of these viruses.

## Conclusion

The seroprevalence of anti-HSV-1 IgG, anti-HSV-2 IgG and anti-EBV IgG decreases in later birth cohorts. This indicates an overall trend of declining risk of getting infected as a child and adolescent. Healthcare implications include a higher proportion of the population being susceptible to primary infection at adult age.

## Data Availability

The datasets used and analyzed during the current study are available from the corresponding author on reasonable request.

## Abbreviations

HHV: Human herpesvirus
IgG: Immunoglobulin G
HSV: *Herpes simplex virus*
EBV: Epstein-Barr virus
CMV: Cytomegalovirus
ELISA: Enzyme Linked Immunosorbent Assay
VZV: *Varicella zoster virus*
STD: Sexually transmitted disease
